# Expression of concern: DDP-resistant ovarian cancer cells-derived exosomal microRNA-30a-5p reduces the resistance of ovarian cancer cells to DDP

**DOI:** 10.1098/rsob.210301

**Published:** 2021-10-21

**Authors:** Ronghua Liu, Yucan Zhang, Peiwen Sun, Changxiu Wang

*Open Biol.* **10**, 20190173. (Published online 29 April 2020) (doi:10.1098/rsob.190173)

Subsequent to the publication of ‘DDP-resistant ovarian cancer cells-derived exosomal microRNA-30a-5p reduces the resistance of ovarian cancer cells to DDP’ Open Biol. 10, 20140281 (published 29 April 2020 (doi.org/10.1098/rsob.190173)), it has been brought to the attention of the Editorial team that there are concerns regarding the validity of the data used in the study.

We are therefore issuing an expression of concern and will notify readers as to the results of our investigation as soon as possible.

Prof. Jon Pines FRS

Editor-in-Chief, Open Biology

